# Methodology for laboratory-based antimicrobial resistance surveillance in animals

**DOI:** 10.14202/vetworld.2022.1066-1079

**Published:** 2022-04-25

**Authors:** Md. Al Amin, Monirul Haque Pasha, M. Nazmul Hoque, Amam Zonaed Siddiki, Sukumar Saha, Md. Mostofa Kamal

**Affiliations:** 1 Quality Control Laboratory, Department of Livestock Services, Savar, Dhaka-1341, Bangladesh; 2 Bangladesh Accreditation Board, Dhaka-1000, Bangladesh; 3 Department of Gynecology, Obstetrics and Reproductive Health, Faculty of Veterinary Medicine and Animal Science, Bangabandhu Sheikh Mujibur Rahman Agricultural University, Gazipur-1706, Bangladesh; 4 Department of Pathology and Parasitology, Chittagong Veterinary and Animal Sciences University, Chittagong, Bangladesh; 5 Department of Microbiology and Hygiene, Bangladesh Agricultural University, Mymensingh-2202, Bangladesh

**Keywords:** animal health, antimicrobial resistance, antibiotic susceptibility test, laboratory, methodology, surveillance

## Abstract

Antimicrobial resistance (AMR) is a crucial and emerging multifactorial “One Health” problem involving human and animal health, agriculture, aquaculture, and environment; and posing a potential public health hazard globally. The containment of AMR justifies effective surveillance programs to explicate the magnitude of the problem across the contributing sectors. Laboratory-based AMR testing and characterization is the key component of an AMR surveillance program. An AMR surveillance program should have a “top management” for fund mobilization, planning, formulating, and multilateral coordinating of the surveillance activities. The top management should identify competent participating laboratories to form a network comprising a reference laboratory and an adequate number of sentinel laboratories. The responsibilities of the reference laboratory include the development of standardized test methods for ensuring quality and homogeneity of surveillance activities, providing training to the laboratory personnel, and in-depth AMR characterization. The sentinel laboratories will take the responsibilities of receiving samples, isolation and identification of microbes, and initial AMR characterization. The sentinel laboratories will use simple antimicrobial susceptibility test (AST) methods such as disk diffusion tests, whereas the reference laboratories should use automated quantitative AST methods as well as advanced molecular methods to explicit AMR emergence mechanisms. Standard guidelines set by Clinical Laboratory Standards Institute or the European Committee on Antimicrobial Susceptibility Testing, should be followed to bring about conformity and harmonization in the AST procedures. AMR surveillance program in animals is eventually similar to that in human health with the exception is that veterinary antibiotics and veterinary pathogens should be given preference here. Hence, the review study was envisaged to look deep into the structure of the AMR surveillance program with significance on laboratory-based AMR testing and characterization methods.

## Introduction

Antimicrobials are small molecules that can inhibit or kill bacteria, but some bacteria can grow and survive despite antimicrobial pressures, a property known as antimicrobial resistance (AMR). Antibiotics are being used for several decades to tackle infections by pathogenic microorganisms in humans, animals, and plants [1]. Alongside the therapeutic use, the non-therapeutic use of antibiotics for food animal production is also remarkable [2]. The emergence and spread of AMR have usually been attributed to the continuous use of antibiotics as therapeutic drugs in human and animal healthcare or as growth promoters in veterinary husbandry [1]. The magnitude of resistance is so versatile that it is observed against nearly all antimicrobials, including so-called last-resort ones used in life-threatening, multidrug-resistant infections [3]. The development of AMR to such an extent has narrowed down the scope of the potential use of antibiotics for the treatment of infections in humans and animals [2]. Moreover, increasing concern over the possibility of AMR transmission through food chains has turned into to a food safety issue. Thus, AMR is considered one of the greatest threats to human health security and is an emerging serious concern to public health, animal health, and food safety authorities [4].

Global and national concerned organizations have categorized the AMR issue as an imminent hazard and have unanimously agreed that tracking the emergence and prevalence of AMR is crucial to minimize the threat to public health [5,6]. Recognizing the urgent need for multispectral action to address AMR, the World Health Organization (WHO) developed a global action plan (GAP) in 2015 for containing AMR through the “One Health” approach. The Food and Agriculture Organization of the United Nations and the World Organization for Animal Health also endorsed complementary plans and strategies for the same purpose. Based on the WHO GAP guidelines, individual countries are adopting and implementing national action plans for the containment of AMR in human, animal, and environment sectors [2]. Surveillance is the key component of the control strategy for the containment of AMR in multiple sectors [7]. AMR surveillance is crucial for the acquisition of information on the extent of the disease burden caused by resistant pathogens, their impact on patient outcomes and patient populations, assessing medical needs, and establishing treatment protocols [8]. Based on the concept of the “One Health” approach, AMR surveillance should be in a holistic way involving both humans and animals [9]. Beyond humans, billions of pets, livestock, and fish depend on antimicrobials, whether as therapeutic or prophylactic agents or as growth promoters are contributing to the emergence and spread of AMR in both animal and human pathogens [10]. The association between antibiotic usage in animals and the development of resistance in commensal bacteria has already been elucidated, rather the transmission of resistant bacteria from food-producing animals to humans has attracted more and more concern [11]. Therefore, AMR surveillance in animals is important to explicit the magnitude and trends of the hazard lying in the sector.

Laboratory-based AMR surveillance starts on the receipt of samples (considering acceptance/rejection criteria) in the laboratory and involves isolation of bacteria and performing antimicrobial susceptibility tests (ASTs). Among the ASTs, disk diffusion and broth dilution methods are usually used in most laboratories for phenotypic AMR characterization [12]. Along with these traditional AST methods, various rapid and efficient methods are being evolved and used in laboratories to study phenotypic AMR characteristics [13-15]. Besides the phenotypic characterization, molecular or genotypic ASTs such as polymerase chain reaction (PCR), DNA microarray and DNA chips, and loop-mediated isothermal amplification (LAMP) are also in use in laboratories for the detection of AMR [13,16]. Recently whole-genome sequencing (WGS) and whole metagenome sequencing (WMS) have shown the potential for deep AMR characterization among microbial communities. Both the methods provide insights into the genetic basis of resistance mechanisms and pathogen evolution and population dynamics at different spatial and temporal scales [17-19]. Although WGS and WMS have the potential for deep AMR characterization, these methods can not differentiate between viable and non-viable bacteria or between pathogenic and non-pathogenic bacteria. Moreover, these methods are unable to generate quantitative AMR data such as minimum inhibitory concentration (MIC) values which are very important for therapeutic purposes [17]. Thus, these novel techniques may not fully replace the conventional “AMR” detection methods in the future.

Hence, the review study was envisaged to look deep into the structure of the AMR surveillance program with significance on laboratory-based AMR testing and characterization methods.

## Literature Search

We conducted literature searches in *Google Scholar*, *PubMed*, *ResearchGate*, and *Crossref* databases. Our research question was “what are the methods used for AMR surveillance in animals?” and the databases were searched using the phrases “AMR surveillance in animals,” “Methods of AMR surveillance in animals,” “Animals and/AMR surveillance.” Searches were filtered for research or review articles published in the English language from January 2011 to June 2021. Grey materials/unpublished documents were searched using *Google* and retrieved from the relevant institutional websites. The articles describing sampling procedure, AST methods, and molecular methods for AMR characterization were considered for inclusion in the review, whereas articles on AMR surveillance methodologies in humans, agriculture, aquaculture, and environment were excluded from the study.

## Selection of Laboratories

The selection of competent laboratories to form a network is crucial for the successful implementation of AMR surveillance programs in animals. This network should include regional or sentinel as well as one or more reference laboratories [20]. There should be top management to coordinate the laboratory activities. The main objective of establishing this laboratory network is to bring about conformity and harmonization in the AST procedures. The top management of a laboratory-based AMR surveillance program will ensure this conformity and harmonization by formulating standard operating procedures (SOPs) based on the Clinical Laboratory Standards Institute (CLSI) or European Committee on Antimicrobial Susceptibility Testing (EUCAST) guidelines [7,17,21]. Other responsibilities of the top management include ensuring necessary resources such as adequate and competent laboratory personnel, suitable environment and facilities, and necessary equipment for efficient execution of ASTs, in addition to assuring the validity of results and controlling the data [22]. The reference laboratories will perform phenotypic and genotypic ASTs on selected antibiotic-resistant isolates received from sentinel labs and look deep into AMR development mechanisms. The reference laboratories will also establish a repository for AMR-relevant isolates. Eventually, the reference laboratories will act as nodal centers for the collaborating laboratories in the network and evaluate the performance of those through arranging Inter-Laboratory Comparisons [23]. The responsibilities of the sentinel laboratories include receiving training from reference laboratories, sample receiving from fields, isolation, and identification (ID) of bacteria, performing ASTs, and storing of bacterial isolates. In addition, sentinel laboratories will also transport predefined drug-sensitive and drug-resistant isolates to the reference laboratories [4].

## Selection of Animal Species

All possible animal species should be included in the AMR surveillance program to achieve the general objectives of establishing baseline data on the prevalence of drug-resistant bacteria among animals. The animal species usually targeted for inclusion in AMR surveillance programs are divided into companion animals, performance animals, food production animals, exotics, and wildlife [21]. Among the companion animals, dogs and cats are selected because of their close association with humans. Similarly, horses are usually selected from performance animals. Food production animals are selected based on their abundance, standards of production systems, farming density, and economic importance. Cattle, pigs, and poultry are food-producing animals frequently considered to be included in AMR surveillance programs. The previous AMR data in microbiota, specifically in zoonotic microbes of an animal population is also an important determinant for inclusion in a surveillance program. Along with the animals from farms and abattoirs, animal-derived products from retail stores should be surveyed for AMR pathogens [4].

## Selection of Bacterial Species

WHO has prioritized a list of pathogens for antimicrobial susceptibility testing in the AMR surveillance program among humans which are *Escherichia coli*, *Klebsiella pneumoniae*, *Staphylococcus aureus*, *Streptococcus pneumoniae*, *Acinetobacter baumannii*, *Salmonella* spp., *Shigella* spp., and *Neisseria gonorrhoeae* [20]. However, bacterial species to be included in AMR surveillance in animals usually differ from pathogens of human interests. Veterinary surveillance programs may not necessarily include bacteria of human interest, although AMR information on such organisms might be valuable. Usually, four distinct categories of bacteria are considered in AMR monitoring programs in animals. This includes animal-only pathogens, zooanthroponotic pathogens, zoonotic foodborne pathogens, and indicator commensal bacteria (Table-1) [11,21,24-27]. Under the animal-only pathogens, methicillin-resistant *Staphylococcus pseudintermedius* is the major companion animals associated bacteria requiring monitoring for AMR. For AMR surveillance in cattle, *Mannheimia haemolytica*, *Pasteurella multocida*, and *Histophilus somni* are included traditionally in surveillance programs [24]. The two main foodborne zoonotic pathogens screened in AMR surveillance programs are *Salmonella* spp. and *Campylobacter* spp. For monitoring of AMR in healthy livestock and poultry, surveillance is usually conducted in indicator bacteria as these organisms are ubiquitously distributed in nature, food, animals, and humans and reflect AMR characteristics arising from selective pressure across these environments. *Enterococcus* spp. and *E. coli* are as such indicator bacteria commonly included in AMR surveillance programs in animals [21,28]. The selection of other bacteria depends on the epidemiology of diseases in the area, which might change over time.

**Table 1:** Bacterial species for inclusion in AMR surveillance programs in animals.

Bacterial Species	Category	Associated Animal Species	Reference
Methicillin-resistant *Staphylococcus pseudintermedius*	Animal-only pathogens	Companion animals	[25]
*Mannheimia haemolytica*, *Pasteurella multocida*, and *Histophilus somni*	Animal-only pathogens	Cattle	[24]
*Actinobacillus pleuropneumoniae, Haemophilus parasuis*	Animal-only pathogens	Pig	[26]
*Pasteurella multocida*	Animal-only pathogens	Cattle, pig	[21]
Enterotoxigenic *E. coli*	Animal-only pathogens	Pig, calves	[21]
*Salmonella* spp.	Animal-only pathogens	Slaughtered food animals	[21]
Methicillin-resistant *Staphylococcus aureus* and extraintestinal pathogenic *E. coli*	Zooanthroponotic pathogens	Dog, cat, horse	[21]
*Salmonella* spp. and *Campylobacter* spp.	Zoonotic foodborne pathogens	Cattle, pig and poultry	[21]
*Enterococcus spp.* and *E. coli*	Indicator bacteria	Healthy livestock and poultry	[27]

## Sample and Sampling

For surveillance programs in healthy livestock feces samples are collected to monitor AMR in *Enterococcus* spp. and *E. coli*; and nasopharyngeal swabs for *M. haemolytica* as well as other respiratory bacterial species [29,30]. AMR surveillance samples for birds include cloacal swabs from adult birds and meconium from chicks. Environmental samples such as farm floor swabs, feed, and drinking water may also be collected [31]. Clinical samples for AMR study are collected from diseased animals and the type of samples depends on the nature and organs or tissues involved in the disease [30]. For monitoring AMR in the food chain, in addition to sampling from animals and farm environment, post-slaughter individual animal cecal contents, carcass rinsates, carcass swabs, ground products, meat juice, lymph nodes, and retail meat cut samples are also collected [32].

A statistically valid number of farms, pens, or individual animals are randomly selected to enroll in the program depending on the AMR surveillance strategy. Alternatively, 30% of farms or pens in a defined area may be selected for sampling, and within each farm or pen 10% of all animals may be randomly sampled for AMR surveillance. Enrolled farms, pens, or individual animals are usually sampled once or more than once over the course of the study [29,33].

Fecal samples are collected per-rectum in the case of an individual animal. A minimum of 4 g of feces is placed in a vial containing modified Cary Blair transport media. Composite fecal samples are collected from floors of pens using a sterile plastic spoon by placing approximately 0.5-1 g of feces into a sterile plastic container. The composite sample is mixed thoroughly, and approximately 4 g of feces from each container is then transferred into a vial containing modified Cary Blair transport media. Nasopharyngeal samples are collected from the deep pharynx using commercially available sterile swabs [29]. After collection, all types of swab sticks are broken into a Cary Blair transport media tube and preserved [29,31].

The collected samples shall be properly labeled with the date of collection, farm or pen number, and the individual ID number for each sample [29]. The samples must be refrigerated in a chilled cooler and transported to the microbiology laboratory in the possible shortest time. On arrival at the laboratory, it is recommended to process the samples immediately for target bacteria isolation, but may be stored short time, not exceeding 6 h at 4°C in a refrigerator [31].

## Bacteria Isolation, ID, and Storage

Based on the type of samples, different recovery methods can be used for the isolation and ID of bacteria. On arrival at the laboratory, the samples must be processed following a standard protocol for the recovery of the target bacteria [3]. The reference method described in any respective ISO standard or in any internationally accepted manual such as Food and Drug Administration (FDA)’s Bacteriological Analytical Manual, or published in any reputed scientific journal should be used [31,34,35]. Bacteria isolation and ID are accomplished in several steps starting from sample preparation followed by pre-enrichment, enrichment/selective enrichment, inoculation to isolation agar, and biochemical tests for the confirmatory ID of bacterial isolates [36]. Various types of pre-enrichment, enrichment/selective enrichment, and isolation agar media are used at different incubation temperatures depending on the type and species of bacteria (Table-2) [29,37-41]. Biochemical tests and test reagents also vary with species of bacteria. In addition to biochemical tests or as an alternative, molecular methods such as conventional and real-time PCR (RT-PCR) can be used for the ID and confirmation of bacteria. Recently matrix-assisted laser desorption/ionization-time of flight mass spectrometry-based methods have been developed and validated for more rapid and confirmatory ID of bacterial isolates [42,43].

**Table 2:** Media used for culture of bacteria recommended for AMR surveillance programs in animals.

Bacterial Species	Pre-enrichment		Selective enrichment		Isolation		Reference
		
	Media	Incubation	Media	Incubation	Media	Incubation	
*Staphylococcus* spp.	-	-	-	-	Baird-Parker agar	35-37°C (45-48 h)	[38]
*Mannheimia haemolytica*	-	-	-	-	Blood agar	37°C (24 h)	[29]
*Pasteurella multocida*	-	-	-	-	Blood agar	37°C (24 h)	[29]
*Escherichia coli*	Lactose broth	35±2°C (24 h)	EC broth	44.5°C (24 h)	L-EMB, EMB agar	35±2°C (24 h)	[39]
*Salmonella* spp.	Lactose broth	35°C (24 h)	TT broth	35±2.0°C (24 h)	XLD agar	35°C (24 h)	[40]
	BPW	35±2°C (24 h)	RV broth	42°C (24 h)	BS agar	35°C (24 h)	
					HE agar	35°C (24 h)	
*Campylobacter* spp.	Bolton broth	37°C (4 h)	Bolton broth	42°C (48 h)	mCCDA	37-42°C (24-48 h)	[41]
					AHB agar	37-42°C (24-48 h)	

BPW=Buffered peptone water, TT=Tetrathionate, RV=Rappaport-Vassiliadis, L-EMB=Levine’s eosin-methylene blue, EMB=Eosin-methylene blue, XLD=Xylose lysine desoxycholate, BS=Bismuth sulfite, HE=Hektoen enteric, mCCDA=Modified campy blood-free agar, AHB=Abeyta-Hunt-Bark

The isolated bacterial strains are preserved in 20-30% glycerol at –80°C temperature until further phenotypic and genotypic AMR characterization is conducted [29]. Isolates may also be stored in nutrient broth containing 50% (v/v) glycerol at –20°C [34].

## Selection of Antimicrobials

Antimicrobial drugs are selected in AMR surveillance programs depending on their present and history of usage in animals, clinical and epidemiological importance, target organisms, as well as the previous reports on the development of resistance against the drugs. Reports on the evolution of new mechanisms of resistance along with the development of resistance against new drugs also influence the selection of antimicrobials. The future concern of the drugs, specifically critical importance to human health, is also an inclusion criterion in surveillance programs [21,32]. The selected antimicrobials that are included to be screened can vary considerably between programs, years, and countries. The antibiotics used in AMR testing in veterinary surveillance considerably vary from those in human surveillance programs. Veterinary surveillance programs usually do not include antibiotics that are relevant for human medicine, although such inclusion might be valuable. Some veterinary surveillance programs include last-resort antibiotics like imipenem, meropenem, and colistin to monitor the animal-associated AMR related public health hazards (Table-3) [11,21,28,29]. However, most antibiotics of human interest are covered by the use of related antibiotics such as flucloxacillin by amoxicillin; cefaclor by cefazolin or cefalexin; ceftriaxone and ceftibuten by cefotaxime; ofloxacin and moxifloxacin by ciprofloxacin; azithromycin and clarithromycin by erythromycin; and tigecycline by minocycline [21].

**Table 3:** Suggested antimicrobials for inclusion in AMR surveillance programs in animals.

Antibiotic class	Antibiotic	Target bacterial species	Reference
Aminoglycosides	Gentamicin	*Salmonella*, *E. coli*, *Campylobacter*, *Enterococcus*, *Staphylococcus*	[11,21,28,29]
	Streptomycin	*Campylobacter*, *Enterococcus*	
Amphenicols	Chloramphenicol	*Salmonella*, *E. coli*, *Enterococcus*, *Staphylococcus*	
Second generation cephalosporins	Cefoxitin	*Salmonella*, *E. coli*, *Staphylococcus*	
Third generation cephalosporins	Cefatoxime	*Salmonella*, *E. coli*	
	Ceftriaxone	*Salmonella*, *E. coli*	
	Ceftazidime	*Salmonella*, *E. coli*	
Fourth generation cephalosporins	Cefepime	*Salmonella*, *E. coli*	
Glycopeptides	Vancomycin	*Enterococcus*, *Staphylococcus*	
	Teicoplanin	*Enterococcus*	
Glycylcyclines	Tigecycline	*Salmonella*, *E. coli, Enterococcus*	
Lincosamides	Clindamycin	*Salmonella*, *E. coli, Staphylococcus*	
Macrolides	Azithromycin	*Salmonella*, *E. coli*	
	Erythromycin	*Campylobacter*, *Enterococcus*, *Staphylococcus*	
Nitrofurans	Nitrofurantoin	*Salmonella*, *E. coli, Enterococcus*	
Oxazolidinones	Linezolid	*Staphylococcus*	
Penicillins	Penicillin	*Staphylococcus*	
	Ampicillin	*Salmonella*, *E. coli*, *Campylobacter*, *Enterococcus*, *Staphylococcus*	
	Amoxicillin	*Salmonella*, *E. coli*	
	Temocillin	*Salmonella*, *E. coli*	
Quinolones	Ciprofloxacin	*Salmonella*, *E. coli*, *Campylobacter*, *Enterococcus*, *Staphylococcus*	
	Nalidixic acid	*Salmonella*, *E. coli*, *Campylobacter*	
	Pefloxacin	*Salmonella*, *E. coli*	
Rifamycins	Rifampicin	*Staphylococcus*	
Sulfonamides	Sulfisoxazole	*Salmonella*, *E. coli, Staphylococcus*	
	Trimethoprim- sulfamethoxazole	*Salmonella*, *E. coli, Staphylococcus*	
Trimethoprim	Trimethoprim	*Salmonella*, *E. coli, Staphylococcus*	
Tetracyclines	Tetracycline	*Salmonella*, *E. coli*, *Campylobacter*, *Enterococcus*, *Staphylococcus*	
	Doxycycline	*Campylobacter*	
Carbapenems	Imipenem	*Salmonella*, *E. coli*	
	Meropenem	*Salmonella*, *E. coli*	
Polymyxins	Colistin	*Salmonella*, *E. coli*	

E. coli=Escherichia coli

## Selection of AMR Test Methods

AMR surveillance in animal health currently relies largely on the isolation of indicator and clinical microorganisms from livestock, environmental, and food samples followed by the phenotypic AMR characterization of the isolates [44]. This culture-dependent phenotypic AMR analysis is sometimes coupled with PCR-based genotypic tests of recovered isolates to explore the molecular basis behind the development of AMR [34]. Although this approach is still very effective in AMR surveillance and is being used extensively in molecular epidemiology studies involving resistant strains from various sources, it has some limitations. This approach does not provide complete information on the mechanisms exerting AMR, or on the presence or spread of AMR genes throughout the animal-originated food production chain. WGS and metagenomics have the potential to be used as powerful tools for in-depth AMR surveillance studies [17].

## Phenotypic Methods

Both qualitative and quantitative phenotypic ASTs are used in AMR surveillance. Disk diffusion test is the commonly used qualitative AST method. Quantitative phenotypic AST methods include broth and agar dilution, Etes^t®^ (bioMérieux, France), and various commercially available semi-automated and automated systems (Table-4) [12,45,46].

**Table 4:** Phenotypic antimicrobial susceptibility test methods commonly used in laboratories.

Name of the AST	Nature of the AST	Media used	Time required (h)	Antibiotics that can be tested	Reference
Disk diffusion method	Phenotypic, qualitative	MHA	18-24	All antibiotics except colistin	[12,46]
Broth dilution method	Phenotypic, manual and quantitative	MHB	24	All antibiotics	[12,45,46]
Agar dilution method	Phenotypic, manual and quantitative	MHA	24	All antibiotics except colistin and sulfa drugs	[45]
Etest^®^	Phenotypic, manual and quantitative	MHA	24	All antibiotics	[46]
Sensitire™	Phenotypic, automated and quantitative	Test panels	18-24	All antibiotics	[46]
Vitek 2^®^	Phenotypic, automated and quantitative	AST cards	4-10	All antibiotics	[46]
BD Phoenix™	Phenotypic, automated and quantitative	Micro-well panels	6-16	All antibiotics	[46]
MicroScan^®^	Phenotypic, automated and quantitative	Panel modules	4.5-7	All antibiotics	[46]

MHA=Mueller-Hinton Agar, MHB=Mueller-Hinton Broth, AST=Aspartate aminotransferase

## Qualitative Phenotypic AST Methods

### Disk diffusion method

Disk diffusion methods measure bacterial growth inhibition zone around paper disks impregnated with a specific concentration of the target drug on agar plates. The Kirby–Bauer disk diffusion assay is widely used to characterize AMR for bacterial isolates in surveillance programs [47]. Disk diffusion methods require prior isolation of bacteria that be spread across the surface of an agar plate. The media used in Kirby–Bauer testing is Mueller-Hinton agar (MHA) prepared at 4.0±0.5 mm depth on either 90 mm or 100 mm or 150 mm Petri dishes. The pH level of the agar is maintained between 7.2 and 7.4. Bacterial inoculum is prepared by suspending colonies in sterile saline to the density of a 0.5 McFarland turbidity standard, approximately corresponding to 1-2×10^8^ colony-forming unit (CFU)/mL for *E. coli*. Inoculum is then spread across MHA plates to form bacterial lawn, followed by placement of the antibiotic-impregnated disks on top of the bacteria. Plates are then incubated overnight, usually at 35°C [48].

The antibiotics diffuse from the disks and form a gradient with the target antibiotic compound into the agar. The inoculated bacteria will grow on the agar to an extent to which the drug is concentrated enough to inhibit the growth. The diameter of the inhibition zone is measured in millimeters either manually or using an automated machine and then is compared with standardized CLSI or EUCAST interpretive criteria to characterize the isolate as sensitive (S), intermediate (I), or resistant (R) to the antibiotic [12].

### Quantitative Phenotypic AST Methods

Quantitative phenotypic AST methods are used to determine the MIC of antibiotics against bacterial isolates. MIC is the lowest concentration of an antibiotic that prevents the visible growth of bacteria and is often expressed in micrograms per milliliter or milligrams per liter [21].

### Broth dilution method

In broth dilution methods, a single bacterial isolate is incubated at 35±1°C for 18-24 h with sequential 2-fold dilutions of the target antibiotics [45]. This test can be accomplished by both broth macro-dilution and micro-dilution methods. MIC is calculated by measuring the optical density (OD) of the broth, with the lowest concentration of each antibiotic that inhibits the visible growth of bacteria. The ranges in antibiotic concentrations tested vary with the type of antibiotic and bacteria but must include the concentration used to define the organism as susceptible or resistant [49]. This procedure is usually performed manually on microplates and can also be performed through many automated and high-throughput platforms [12]. Mueller–Hinton broth (MHB) is used as a diluent medium for antibiotics. In some cases, additional supplements such as 5% lysed horse blood, hemin (5 mg/mL), Vitamin K (1 mg/mL), or other compounds may be required depending on the type of bacteria or antibiotics [45].

For bacterial inoculum preparation in broth dilution methods, morphologically similar colonies cultured overnight on a nonselective solid medium are suspended in MHB medium and adjusted to 5×10^5^ CFU/mL. For obtaining the desired bacterial concentration, a suspension of 0.5 McFarland is prepared first and diluted 100× to reach a density of 10^6^ CFU/mL by adding 0.1 ml 0.5 McFarland suspensions to 9.9 mL broth. Finally, an equal volume of bacterial inoculum is added with an equal volume of a liquid medium with antibiotics in test tubes (macro-dilution) or wells of a microtiter plate (micro-dilution) [50].

### Agar dilution method

The agar dilution method is similar to broth dilution in principle with the exception that; two-fold serial dilutions of the target antibiotic are added to melted agar before solidification. The inoculum is prepared to obtain a final concentration of 1×10^4^ CFU/spot by diluting the 0.5 McFarland suspension 10× in NACL or MHB and spotting 1 mL of such suspension on the MHA media with appropriate antibiotic dilutions [51]. The prepared plate was incubated at 35±1°C for 18-24 h for growth and the MIC was estimated from the lowest concentration of each antibiotic that inhibits visible growth [46].

In both broth and agar dilution methods, dissolutions of antibiotics are also required for MIC determination. Several types of solvents such as water for most beta-lactams, fluoroquinolones, and aminoglycosides; alcohol for macrolides, chloramphenicol, and rifampicin; dimethyl sulfoxide for carbapenems are used for preparing stock solutions of antibiotics [45].

### Etest^®^

Etest^®^ is an assay for simultaneous determination of antimicrobial sensitivity as well as MIC value [16]. This combined qualitative and quantitative method has been developed and marketed by bioMérieux. In this procedure, antimicrobial sensitivity and MIC are determined by placing a plastic strip impregnated with a gradient of a specified antibiotic onto an agar plate. Subsequently, the agar plate is inoculated with the test isolate of bacteria and incubated for 18-24 h. After incubation, the bacterial growth becomes visible and a symmetrical inhibition ellipse centered along the strip is observed. The MIC value is calculated from the scale in terms of mg/mL where the ellipse edge intersects the strip. The media usually used for the method are MHA for aerobes and Brucella blood agar for anaerobes [12]. Etest^®^ can be used to test multiple antibiotics per plate against only one organism and the time required to yield results is comparable to other agar diffusion or dilution methods [46].

### Automated methods

Uncertainties in result interpretation, labor intensiveness, and long AST profiling time have driven the laboratories to use automated systems to determine antibiotic susceptibility profiles. There are several commercial automated MIC determination systems are currently available. Among these, the following four systems have been approved by FDA: Sensitire™ (Trek Diagnostic Systems, Thermo Fisher Scientific, USA), Vitek 2^®^ (bioMérieux), MicroScan^®^ (Beckman-Coulter, USA), and Phoenix™ (BD, Canada). These systems are reliable, easy to use, and can be integrated with laboratory information management systems (LIMS) [46].

### Sensitire™

Sensitire™ is based on the broth microdilution method and the actual detection of bacterial growth can be read through a fully automated approach using fluorescence technology. The specific enzymes produced by the organism over the overnight incubation period cleave the bond between the fluorophore and the quencher substrate; release the fluorophore to emit fluorescence [52]. The amount of fluorescence is directly proportionate to the growth of the organism and is used to report the MIC values. This fully automated system is capable of handling multiple samples with 96-well microdilution plates that can be inoculated with a Sensititre Autoinculator. Test panels are available for both Gram-positive and Gram-negative bacteria [53].

## Vitek 2^®^

Vitek 2^®^ system utilizes the broth microdilution technique and is used simultaneously for bacterial ID and AST profiling [46]. This system uses “AST cards” which contain microwells with fluidic connections to an automatic sample loading device. AST cards contain 64 microwells each of which is loaded with dehydrated bacterial culture media and antibiotics at different concentrations. One well in the cards contains only dehydrated culture media without any antibiotic and is used as positive control well. This fully automated system uses attenuation of light measured by an optical scanner for growth or no growth detection [54]. For the test assay, a colony of the target bacterial isolate is first suspended in a vial using sterile saline solution and adjusted to 10^8^ CFU/mL; henceforth, the vial is coupled with an AST card, scanned, and placed into the VITEK^®^ system. The suspension is finally diluted automatically to 5×10^5^ CFU/mL, loaded into the AST cards, sealed, and incubated within the VITEK^®^ system. The bacteria are incubated for 18-24 h and yeast for 36 h with periodic growth monitoring. The MIC values are calculated from the OD values in individual wells arising from bacterial growth or no growth, and a MIC table for different antibiotics. A report, along with its interpretation, is generated automatically by the system [46].

## Phoenix™ (BD) Automated ID and Susceptibility Testing System

BD Phoenix™ is a microdilution-based automated system used for simultaneous bacterial ID and antibiotic susceptibility testing [46]. The system comprises micro-well panels and each panel contains an ID and an AST section, each with multiple microwells. The AST section of the panels consists of 84 wells including one well for positive control. The growth of bacteria in the micro-wells is detected using a redox indicator. Each microwell in the panels is pre-loaded with an antibiotic at a particular concentration and test assays are prepared by rehydrating the antibiotics with the addition of test bacterial suspension. The panels are incubated over 4-16 h depending on the type of microorganisms and scanned for microbial growth using chromogenic or fluorogenic substrates to obtain MIC values. The BD Phoenix system is capable of reading 99 AST panels at a time using a dedicated expert software to report MIC value for a given antibiotic along with susceptible, resistance, or intermediate interpretation [55].

## Micro-scan Walk Away^®^

Micro-scan walk away**^®^** is also an automated system for bacterial ID and AST based on broth microdilution method and capable of medium and large-scale operations using 40 and 96-panel modules, respectively. Like other automated systems, test assays are prepared by rehydrating dried antibiotics and media in the wells by inoculating the bacterial suspension. Test assays are incubated for 4.5-18 h and growth or no growth of bacteria in individual wells are determined by colorimetric readings. The threshold concentration for bacterial detection in this system is 2×10^7^ CFU/mL [46].

## Quality Control in Phenotypic ASTs

Quality control should be carried out in each of the phenotypic AST methods following standardized SOPs, controlling medium sterility, using reference strains, and obtaining quality test results. The reference bacterial strains recommended by either CLSI or EUCAST must be used in each test (Table-5) [29,45,48,56,57]. The AST findings must be matched with the CLSI or EUCAST interpretive criteria to report the isolates’ AMR characteristics. The inhibition zone diameter or MIC values of the tested antibiotic for reference strains should be within the range recommended by EUCAST and CLSI [45]. CLSI has determined clinical breakpoints for registered veterinary and human antimicrobial agents using in vitro and in vivo data to predict the likelihood of clinical cure based on pharmacokinetics and pharmacodynamic parameters. EUCAST has developed clinical breakpoints for human pathogens and those for veterinary pathogens are still under development. The clinical breakpoints are not available for all antimicrobials and animal species; thus, human clinical breakpoints should be used if veterinary-specific breakpoints are not available [21].

**Table 5:** Reference bacterial strains recommended by European Committee on Antimicrobial Susceptibility Testing and Clinical Laboratory Standards Institute for using in ASTs.

Test bacteria	Reference bacteria	AMR characteristics of the reference bacteria	Use	Reference
*Enterobacter* spp*., Escherichia coli, Klebsiella* spp*., Salmonella* spp*.,* *Shigella* spp*., Yersinia* spp*.* and other Enterobacterales	*Escherichia coli* (ATCC 25922)	Non resistant to antibiotics	Used as negative control strain	[29,45,56,57]
	*Escherichia coli* (ATCC 35218)	Beta-lactamase-producing strain	Used as positive control for the assay of beta-lactam antibiotics	
	*Klebsiella pneumoniae* (ATCC 700603)	Beta-lactamase SHV-18 producing strain	Used as positive Control	
*Pseudomonas* spp., *Acinetobacter* spp.	*Pseudomonas* *aeruginosa* (ATCC 27853)	Multidrug resistant strain	Used as positive control	
*Staphylococcus* spp.	*Staphylococcus* *aureus* (ATCC 29213)	Weak beta-lactamase producing and Oxacillin sensitive strain	Used as negative control strain for MIC determination of beta-lactam antibiotics	
*Enterococcus* spp.	*Enterococcus* *faecalis* (ATCC 29212)	Multidrug resistant strain	Used as positive control in ASTs	
*Streptococcus* spp., *Listeria monocytogenes*	*Streptococcus* *pneumoniae* (ATCC 49619)	Moderately penicillin-resistant strain	Used as penicillin susceptible control strain	
*Pasteurella multocida*	*Haemophilus influenza* (ATCC 49766)	Ampicillin-susceptible strain	Used negative control strain for MIC determination of ampicillin	

MIC=Minimum inhibitory concentration, AST=Antibiotic susceptibility test

## Genotypic AMR Characterization Methods

Phenotypic methods for AMR determination are applicable only to the cultivable microbes and incapable of interpreting the mechanisms of emergence and spread of AMR in diverse and complex microbial communities where large fractions are uncultivable bacteria. Genotypic methods can be applied to overcome this limitation and are applicable to both cultivable and uncultivable bacteria. Bacterial AMR is usually genetically encoded and these genetic determinants can be identified and characterized by PCR and gene sequencing technologies [58].

### PCR assay

PCR assay is being used to detect AMR genes in microorganisms through a target-based approach. This method enables the rapid detection of previously known and characterized antibiotic-resistant genes harbored in the microorganisms, thus having the potential to be an important tool for inclusion in AMR surveillance programs. For PCR, DNA from the culture-isolated microorganisms, or even directly from samples is extracted first and used as the template for PCR reaction. Predesigned primers are used to amplify the target region on template DNA [59]. Along with conventional PCR, RT-PCR and LAMP techniques can be employed for AMR surveillance [60]. The wide use of multiplex PCR has made AMR monitoring easier in which several resistance genes can be detected simultaneously with the incorporation of different primers in the same assay mix [61]. The amplicons are visualized either by gel electrophoresis in conventional PCR or by the addition of different dyes in RT-PCR. The AMR genes for which detection PCR is commonly used are *van*A (encoding vancomycin resistance), *mec*A (encoding methicillin resistance), *amp*C (encoding ampicillin resistance), *mcr*-1 and *mcr*-2 (encoding colistin resistance), *ndm-1* (encoding carbapenems resistance), ESBL genes *bla*TEM, *bla*SHV, *bla*CTX-M, *bla*OXA, *bla*VIM, *bla*NDM, and *bla*KPC, etc. [62].

### WGS and WMS methods

The introduction of high throughput sequencing technologies and bioinformatics tools has enabled WGS and WMS as potential methods for rapid species and AMR gene ID and characterization. Both WGS and WMS can be used reliably to predict the phenotypic AMR using readily-available online tools and is suitable to be incorporated in AMR surveillance programs in animals [58,63].

In a standard WGS protocol, DNA is extracted from the isolated target bacteria followed by library preparation by shearing DNA into a pool of fragments. Thereafter, the library is sequenced through a set of sequencing reactions and analyzed in a sequencing machine capable of determining the DNA sequence of each fragment in the library. These fragments are assembled into short contigs, ultimately to the complete genome for further analyses, including gene prediction and annotation, comparative genomics, and evolutionary analysis [19]. WMS technique can be used for the culture-independent analysis of complex microbial communities to generate useful data on AMR genes occurrences. The WMS protocol is the same as the WGS protocol with the exception that DNA is extracted directly from samples here [17].

Both WGS and WMS are based on high throughput next-generation sequencing techniques and have been developed by a number of commercial organizations and operated on different platforms such as the Illumina/Solexa,454/Roche, Ion PGM from Ion Torrent, AB SOLiD System, and Oxford Nanopore MinION. These systems use different chemistry for their operation [64]. The technology has advanced in leaps and bounds with the refinement of bioinformatics platforms and upgradation of hardware since its advent almost two decades ago.

Two common bioinformatic approaches for detecting AMR genes are Basic Local Alignment Search Tool (BLAST)-based analysis of *de novo* assembled draft genome against a reference database and mapping analysis of raw sequencing reads [20]. In assembly-based methods, the *de novo* assembly of the bacterial genome from short-reads is performed by De Bruijn graph-based assemblers such as SPAdes, Velvet, ABySS, and SOAPdenovo. Sequencing reads are divided into shorter overlapping fragments (k-mers) to form a network graph. Henceforth, the assemblers reconstruct the genome sequence by finding an optimum path (Euler’s path) through the graph that visits each edge once. IDBA-UD, MEGAHIT, MetaSPAdes, and MetaVelvet are the assemblers used to assemble metagenomic sequences [58]. Following assembly, AMR gene search is accomplished using homology-based algorithms such as BLAST [65]. In read-based methods, AMR genes in a sample are detected without genome assembly either by aligning reads to the reference databases using pairwise alignment tools such as Bowtie2 or BWA; or by splitting reads into k-mers and mapping them to the reference databases. Read based methods are more sensitive than BLAST-based analysis of draft genomes [58,62].

The reference databases used to identify AMR genes in the genome of an isolate or in metagenome are ResFinder, ABRicate, Search Engine for Antimicrobial Resistance, Comprehensive Antibiotic Resistance Database, Antibiotic Resistance Gene-ANNOTation, Antimicrobial Resistance Identification By Assembly, or Resistance Gene Identifier [62]. Another open-source, publicly available assembly-based hybrid server, Metagenomics Rapid Annotation using Subsystem Technology is also used to study and compare the distribution and relative abundance of clinically significant ARGs across animal metagenomes [66].

## Discussion

AMR is a serious global problem of complex epidemiology as resistant organisms exist in multiple sectors like humans, animals, food, and the environment [67]. The evolution of AMR bacteria in animal production settings represents a potential health hazard for both humans and animals. AMR bacteria of animal origin usually spillover from animals to humans through animal-originated foods, water, or direct contact with animals. Factors such as over-prescription of antimicrobials, feeding low doses of antimicrobials as growth promoters, and using non-approved drugs contribute to the emergence of AMR [2,4]. The containment of AMR problems warrants a comprehensive surveillance program in all contributing sectors through a “One Health” approach [67]. Laboratory tests for the characterization of phenotypic and genotypic AMR properties of bacteria are an integral part of surveillance programs [46]. A competent laboratory network comprising an adequate number of sentinel and reference laboratories along with proficient laboratory personnel, necessary equipment and materials, and standardized test protocols is crucial for the efficient execution of laboratory activities in an AMR surveillance program [23]. The laboratory network in low-income settings is unlikely to have sufficient resources. Thus, flexibility across different systems may be allowed but should ensure sufficient standardization of core protocols to generate valid and comparable data [68].

AMR surveillance programs in the animal health sector should be coordinated by top management. The top management eventually acts in the role of centralized leadership and is responsible for fund mobilization and networking between disciplines and sectors through shared meetings, discussions, and reporting. The top management will analyze the needs of participating labs and respond accordingly [22,23,69]. Setting a pragmatic surveillance program is the main responsibility of the top management. Rational selection of the study area and animal species is critical. Selection of too large study area or too many animal species or bacterial species to be monitored may turn the surveillance program into a clumsy one. In farming settings, animal diseases are usually tried to control by management and vaccination resulting in very sporadic disease outbreaks. Therefore, AMR surveillance implementation in healthy animals, rather than in diseased animals in a clearly defined area would be more rational. AMR monitoring in *E. coli* bacteria in feces samples or *M. haemolytica* in nasopharyngeal swabs are suggested to explicate an in-depth picture of the problem [28-30].

The establishment of a laboratory network engaging a competent central reference laboratory and a sufficient number of sentinel laboratories is another important responsibility of the top management. The central reference laboratory should be identified from existing facilities or be established for AMR surveillance. If such scope does not exist, assistance from a competent laboratory in neighboring countries should be availed through collaborative agreement. The central reference AMR laboratory should be accredited in accordance with global standard ISO/IEC 17025, or be working toward laboratory accreditation for ensuring reliable test results [70]. The sentinel site laboratories should be at a convenient distance from the study area and must have sufficient resources for the execution of vested duties. Adequate training, if required must be provided to the labs of all levels [68,70].

Laboratory-based activities in AMR surveillance programs are eventually started at sentinel laboratories. However, the activities of the sentinel labs should be limited to the sample reception, isolation and ID of target bacteria, and AMR characterization by disk diffusion test. Disk diffusion test is easy to execute and suitable for testing multiple drugs on a single agar plate, and thus can be established in sentinel laboratories with limited-resource settings [46,68].

Central reference laboratory should determine MIC values of the antibiotics through broth or agar dilution methods. Although both broth macro-dilution and micro-dilution methods are easy to interpret and efficient in determining MIC of antibiotics, the broth macro-dilution method is cumbersome, time-consuming, and difficult to run multiple samples simultaneously. Whereas broth micro-dilution method is easier to perform with minimal expertise and enables multiple samples or antibiotics testing at a time; hence, it can be conveniently used in reference laboratories. In the agar dilution method, the preparation of agar plates is cumbersome, but this method is reproducible and enables the efficient testing of large numbers of bacterial isolates [49]. However, the scope of this method is limited by frequent misinterpretation of MIC values and the inability to test certain antimicrobials (Table-4) [46].

Automated MIC determination systems can be used in the reference laboratories if the sample size is too large. However, the scope of using automated systems has been limited by its high cost. Moreover, the systems require a pure isolated culture of bacteria for AST determination and interpretation, which is time-consuming. However, automated systems are user-friendly by their easy and smooth workflow [21]. These systems enable simultaneous large number sample testing and minimize the uncertainties from result interpretation, thus should be incorporated in reference laboratories for the AMR surveillance program.

Genotypic AMR characterization is performed to explicit the genetic determinants of AMR emergence. PCR is used commonly for this purpose, but this method only enables the detection of previously known and characterized antibiotic-resistant genes harbored in the microorganisms. Even, a single nucleotide polymorphism in the AMR gene may mislead the false-negative result [59]. AMR gene sequencing followed by homology search may overcome the drawback of PCR. WGS or WMS based on high throughput next-generation sequencing are the best alternatives to PCR. However, the high cost of the gene sequencing platforms and requirements of deep knowledge in bioinformatics are the main obstacles to the comprehensive use of the methods [17]. However, decreasing costs and increasing rapidity and reliability of sequencing technologies are creating scopes for adopting next-generation sequencing platforms, especially at the reference laboratory level for AMR surveillance programs in animals. Moreover, the development of various online tools and advanced computational algorithms has further simplified the use of next-generation sequencing platforms in AMR surveillance programs [17,19].

## Recommendation

Based on the above discussion, the following recommendations could be followed for setting and executing an AMR surveillance program in animals-

- The AMR surveillance program in animals should be well-structured, comprising top management and a competent laboratory network
- The top management of the AMR surveillance program will identify a competent laboratory network and define a catchment area for surveillance study
- The top management will formulate a pragmatic AMR surveillance program
- The top management should provide support to improve the capacity and capability for AMR surveillance and monitoring at all levels involved, allocate sufficient funds to ensure the availability of consumables, diagnostics, and reagents
- Standardized protocols should be developed by the reference laboratory for the harmonization of all activities at all levels
- The sentinel laboratories will be responsible for sample receiving, bacteria isolation and ID, and performing AST by disk diffusion test
- The reference laboratory will go for in-depth AMR characterization through both phenotypic and genotypic methods using automated AST devices and WGS or WMS approach, respectively
- Appropriate reference bacterial strains must be used for antibiotic susceptibility testing
- Antibiotic susceptibility test reports should be given following internationally accepted CLSI or EUCAST breakpoints
- Evaluate the surveillance methods used and the data collected periodically to ensure that they are serving the purposes; make necessary adjustments to address emerging issues such as emerging pathogens and new commodities.

## Conclusion

AMR is considered one of the most dreadful public health hazards and its surveillance is crucial for the containment of the problem. AMR is eventually a “One Health” problem where the animal sector is a significant contributor to it. Therefore, AMR surveillance in animals is very important for understanding the emergence and magnitude of the burden and thereafter for mitigating the hazard. The surveillance system is also very important to gather data that will increase our knowledge and understanding of the complex mechanism and epidemiology of AMR. This information is crucial for treating infection, making policy recommendations, and developing strategies to reduce the magnitude of AMR burden. This review study explicit the current structure and methodology of an AMR surveillance program used in animals and recommends an efficient and pragmatic surveillance system comprising both phenotypic and genotypic ASTs. This comprehensive review tried to retrieve the maximum available relevant information. Nevertheless, due to the limited open data sharing policy by some of the journals and restricted access to some of the databases, the search may not be exhaustive and the review is limited by the inclusion of a small number of articles. Even though the study sufficiently illustrated the structure, objectives, and methodologies of an AMR surveillance program. Hence, this review article could be used as a reference work to formulate, adopt, and implement an effective and pragmatic AMR surveillance program in veterinary practices.

## Authors’ Contributions

MAA: Conceptualized and designed the review. MAA, MHP, and MNH: Collected the literature. MAA and MNH: Drafted the manuscript. MHP, AZS, SS, and MMK: Edited and finalized the manuscript. All authors have read and approved the final manuscript.
